# Challenges faced by midwives in the implementation of facility-based maternal death reviews in Malawi

**DOI:** 10.1186/s12884-023-05536-2

**Published:** 2023-04-24

**Authors:** Mercy Dokiso Chirwa, Juliet Nyasulu, Lebitsi Modiba, Makombo Ganga- Limando

**Affiliations:** 1 Department of Health Studies, University of South Africa, Preller Street, Po Box 392, Pretoria, 0003 South Africa; 2 Division of Health Systems and Public Health, Department of Global Health, Stellenbosch. University, Cape Town, South Africa

**Keywords:** Maternal deaths, Facility-based maternal death review, Midwife challenges

## Abstract

**Background:**

Maternal death reviews provide an in-depth understanding of the causes of maternal deaths. Midwives are well positioned to contribute to these reviews. Despite midwives’ participation as members of the facility-based maternal death review team, maternal mortality continues to occur, therefore, this study aimed to explore the challenges faced by midwives as they participate in maternal death reviews in the context of the healthcare system in Malawi.

**Methods:**

This was a qualitative exploratory study design. Focus group discussions and individual face-to-face interviews were used to collect data in the study. A total of 40 midwives, who met the inclusion criteria, participated in the study. Data was analyzed manually using a thematic content procedure.

**Results:**

Challenges identified were: knowledge and skill gaps; lack of leadership and accountability; lack of institutional political will and inconsistency in conducting FBMDR, impeding midwives’ effective contribution to the implementation of maternal death review. The possible solutions and recommendations that emerged were need-based knowledge and skills updates, supportive leadership, effective and efficient interdisciplinary work ethics, and sustained availability of material and human resources.

**Conclusion:**

Midwives have the highest potential to contribute to the reduction of maternal deaths. Practice development strategies are required to improve their practice in all the areas they are challenged with.

## Background

Sub-Saharan Africa and South Asia combined account for 86% of all the estimated global maternal deaths [1]. Malawi is one of the countries in the world that did not meet the Millennium Development Goal 5 target of reducing maternal deaths by 75% by 2015 despite a significant increase in antenatal care attendance and institutional deliveries by skilled healthcare workers [1]. In 2016, Malawi’s mortality ratio (MMR), stood at 439/100,000 live births, having decreased from 675/100,000 in 2010 [2]. While the latest, maternal mortality ratio is estimated at 349 per 100,000 live birth [3]. Haemorrhage, hypertension, sepsis, abortion, embolism are the common causes of maternal mortalities [4]. Other factors contributing to the high vulnerability of maternal morbidity and mortality in Malawi and globally are: i) delays seeking care ii) poor referral system, iii) lack of access to essential health supplies, equipment, iv) inadequate financing, leading to low purchasing power v) lack of human resource, coupled with inadequate knowledge, skills and lack of competencies vi) inefficient leadership and governance and vii) inadequate information for making decision [5].

Malawi’s health system is fragile with a critical shortage of human resources for health. Currently, the staff-patient ratio is 2 physicians per 100,000 people and 0.5 nurse/midwives per 1000 patients. The World Health Organisation recommends a nurse/midwife patient ratio of 1 to 175. Current global shortage of midwives is 900,000 [6, 7]. The global Sustainable Development Goal 3 target is calling for governments to reduce maternal deaths to 70/100,000 live births by 2030 and neonatal deaths by 12/1000 live births [8]. Skilled birth attendants play a critical role in making pregnancy safer and saving lives of women and newborns [8]. Malawi is one country in Sub-Saharan Africa that is characterized by a heavy burden of diseases both communicable and non-communicable. The country has one of the highest population densities in the region, the total fertility is at 4.4 with a life expectancy of 39 years. Malawi faces several challenges including inadequate finances to support the poverty reduction program, a high level of illiteracy, and critical incapacities in institutions implementing development programs . The weak healthcare system in Malawi calls for urgent strategies and consented efforts to strengthen the delivery of the expected quality maternal health services and reduce the mortality rates [9]. The nursing and midwifery workforce, as frontline workers, and members of the multidisciplinary team, have a huge role to play in strengthening the global health system through the provision of sustained quality maternal health services [9].

The World Health Organization introduced the Safe Motherhood program to accelerate the reduction of existing high maternal and neonatal mortality in Low-Income Countries (LMIC). Despite this initiative, more than 15 years down the line, women continue to die from direct and indirect preventable pregnancy-related causes globally which could be averted with cost-effective measures [10]. In 2004, the maternal death review was introduced by the World Health Organisation as an alternative initiative to the safe motherhood project to further reduce the escalating maternal deaths globally [11]. A year later, in 2005, Malawi adopted and implemented the facility-based maternal death review (FBMDR) which is a process which involves the following steps: identification of women who die during child birth, collecting detailed data surrounding their death, analysis of the data to find possible causes of maternal death, developing recommendations to address the identified problems, implementing the recommendations, evaluating effectiveness of implemented actions and making necessary refinement to improve the whole maternal health care and prevent avoidable deaths. The FBMDR aims at tracing the passageways of women who die, through the healthcare system and within the healthcare facilities, to ascertain preventable issues that could change and improve maternal care yet to come [12]. The maternal death review is an educational process for health care professionals and students who take care of women during pregnancy, labour and delivery and postnatal period and its related issues. It also serves as a way to make health providers answerable for their actions [11, 12].

A national maternal death review plan and policies were put in place in Malawi. The maternal death review forms were developed, and necessary orientation programs were carried out on how to conduct the reviews. District hospitals in Malawi constitute the pillar of the implementation of the FBMDR process [13]. Both the facility and community-based maternal death reviews are conducted at the district level monthly. The Ministry of Health encourages every hospital to individually review all maternal deaths that occur in the health facilities and the communities. Maternal death notifications are completed by a senior health professional involved in the management of that woman. Within 72 h of the maternal death, they are sent to the Ministry of Health Reproductive Health Unit accompanied by a narrative report, which summarizes the circumstances surrounding the death of the woman. Thereafter, the facility’s maternal death review team conducts a review to ascertain the cause of death. At the end of the review, the chairperson completes the review form, which is submitted to the head of the facility and is later passed on to the district and the regional offices. These reports are reviewed and discussed at the quarterly and yearly regional health review meetings. At the review meetings, the various health institutions (hospitals and district health directorates) present cases of maternal deaths that occurred at their respective institutions. These presentations are followed by discussions that focus on where, when, how, and what interventions should be implemented to prevent avoidable problems [13].

District-based midwives are involved in the implementation of most of the government’s initiatives aimed at improving professional practice and reducing maternal mortality including maternal death review [13, 14]. Midwives as skilled attendants are frontline members of the healthcare workforce who are the first point of contact for women during pre-conception, pregnancy, childbirth, and in the post-delivery. They know the mother’s stories much better than the other members of the healthcare team. They know what mothers are going through, they have lived experiences in their practice providing maternal healthcare. They are aware of their strengths and weaknesses in their competencies. They are therefore well positioned to participate in reviewing circumstances surrounding women’s death as individuals as well as members of the multidisciplinary review team [12]. By participating in the reviews midwives do understand and learn from their inefficiencies, omissions, and what could be done to prevent avoidable maternal deaths. Reviews are an important way of monitoring the quality of the healthcare system that can facilitate the identification of health systems failure and gaps that contribute to women’s deaths [12]. Well-trained midwives who are skilled and continuously participate in professional development and practice development can effectively participate in the reviews and implementation of the recommendations to prevent future maternal deaths [12]. Each maternal death or case of life complication has a story to tell and can provide indications on practical ways of addressing the problem [12]. Global experiences in the use of such approaches have led to the successful implementation of Facility Based Maternal Death Review (FBMDR) at various levels of the healthcare delivery system, including: Individual facility and national levels, influencing health professionals, healthcare planners, and managers working in maternal and newborn health, who strive to provide improved quality of care outcomes [12]. The reviews provide an in-depth investigation into causes of and circumstances surrounding maternal deaths occurring in health facilities in the country [13]. The mechanisms of reviews are widely recommended as interventions to deepen an understanding of the prevalence and incidence of possible causes of maternal mortality [13]. The reviews enable the development of recommended actions for improving the quality of maternal healthcare, which could be key to efforts in the attainment of Sustainable Development Goal 3.1 by 2030, although there is little robust evidence to support this discourse [15, 16]. In Malawi, healthcare providers face many challenges at different levels of implementing FBMDR including: lack of knowledge, skills, and shortages of staff and material resources [13].

Midwives have been participating in the implementation of FBMDR as individual practitioners and as members of multidisciplinary teams at all levels of the healthcare delivery system in the country but the challenges they face in their contribution to the implementation of maternal death review remain obscure [17]. This study was therefore conducted to illuminate these challenges. The results of the study and other studies have, however, revealed several challenges midwives face while contributing to the implementation of the FBMDR, which include shortage of staff, lack of resources, inadequate knowledge and skills, effective leadership gap, and lack of accountability, lack of institutional political will, multidisciplinary team collaboration and inabilities to consistently conduct the review. Midwives’ capabilities to mitigate these challenges, therefore, require consented efforts and support at all levels of the healthcare delivery system, from policy level to program planners, regulatory body, academia, managers, care providers, and the community. The FBMDR provides a timeous opportunity for midwives, hospital health management, and other multidisciplinary team members to learn from circumstances leading to maternal deaths and suggest possible solutions to avert potential future maternal deaths. Monitoring the implementation of recommendations helps to promote accountability for healthcare outcomes amongst multidisciplinary teams. When the results of maternal and perinatal death surveillance and review are acted upon by all concerned parties, the recommendations made from reviews can lead to sustained quality improvement of maternal healthcare services and consequently prevent future maternal mortality ratios [18].

In many low-income countries where FBMDR is being practiced, findings have shown: a lack of knowledge among facility staff; inadequate in-service training, increased workload, limited knowledge of health policies, and difficulties following the procurement act among midwives [19, 20]. The challenge, as with many healthcare system interventions, is to find a way to provide catalytic assistance and strengthen the capacity for Maternal Death Surveillance and Response (MDSR) which is embedded in the healthcare system [12, 19]. Since the initiation of FBMDR in Malawi, not much has been documented to highlight the processes involved including challenges experienced. Therefore, in this paper, we discuss challenges faced by midwives in the implementation of facility-based maternal death reviews in Malawi.

## Methodology

### Study design

A qualitative exploratory descriptive design was used to explore and describe the experiences and views of the midwives working at the district hospitals regarding (i) challenges they face in their contribution to the implementation of facility-based maternal death review and ii) possible solutions and facilitators to enhance practice development of midwives in the implementation of facility-based maternal death review in the context of the healthcare delivery system in Malawi. Focus group discussions (FGD) and individual face-to-face in-depth interviews were conducted with Registered Nurse Midwives and Nurse Midwife Technicians who participated in the implementation of facility-based maternal death reviews (FBMDR) in district hospitals in Malawi.

### Study site and service delivery

Malawi has a total of twenty-six district hospitals serving twenty-eight administrative districts. Each district hospital serves a catchment area of an estimated population of 600,000 to 900,000 on average within a geographical distance of 15 to 20 km. District hospitals in Malawi function as coordinating centres for local health information and planning for programs such as safe motherhood and integrated management of childhood illnesses [21]. The district hospitals operate 24 h per day and 7 days per week. They provide free services for both in and outpatients that include: medical, surgical, reproductive, maternal, and child health services. The reproductive health services offered at district hospitals include: focused antenatal care, labour and delivery, postnatal care, family planning, cancer screening, and management of sexually transmitted infections. In addition, all district hospitals in the country have established maternal death review teams that implement and coordinate all the activities of FBMDR in the district [22].

### Sampling

To ensure maximum variation in the contextual working environment of participants, six districts (3 rural and 3 urban) out of Malawi’s 28 district hospitals across three geographical regions (Northern, Central & Southern) of the country that met the criteria were purposely selected for the study [21].

### Recruitment of study participants

The midwifery workforce in Malawi is composed of two main cadres: the Registered Nurse Midwife who undergoes four years of post-secondary education, including an optional year of midwifery, and exits with a bachelor’s degree in Nursing and a university certificate in midwifery, while Nurse Midwife Technicians are trained at college diploma for three years, which includes two years of general nursing and one year of midwifery training [23]. In the hospital settings Nurse Midwife Technicians practice under Registered Nurse Midwives but, in health centres, the former is often the only nursing midwifery staff available to perform all available medical care including maternal and health care services alongside Medical Assistants who are also prepared a diploma level from medical schools within the country and have minimal midwifery knowledge and skills [23]. All nurse-midwives participating in the FBMDR at district hospitals were eligible for the study. A total of 40 participants were selected for the study using a non-probability sampling technique from the six sampled district hospitals (24 were Registered Nurse Midwives and 16 Nurse Midwives Technicians). The initial sample consisted of thirty participants, but this number was increased to forty at the end of the study to reach data saturation [24]. All methods were carried out under relevant ethics guidelines and regulations and informed consent was sought and signed by all participants. All midwives working in maternity units with a minimum of two years’ work experience (antenatal, labour and delivery, and postnatal), participating in the implementation of facility-based maternal death review for at least one year before data collection and working continuously in the maternity units for the specified period met the inclusion criteria. In this study, triangulating the methods of data collection helped to enrich the data by complementing the participants' views and opinions. Triangulation refers to the use of multiple methods of data collection or data sources. This approach is also viewed as a qualitative strategy to test the validity of convergence of information from different sources to create a more in-depth picture of a research problem and to interrogate different ways of understanding a research problem [24].

### Data collection process

The researcher developed interview guides for individual face-to-face in-depth interviews and focus group discussions as tools to guide data collection. The tools were pilot tested to develop the lines of questioning when probing, to ensure the researcher’s familiarity with its use, and to identify and exclude any ambiguities before initiating data collection. The pilot testing consisted of one focus group interview with five nurse-midwife technicians and three individual face-to-face in-depth interviews with registered nurse-midwives at one hospital, which was not part of the six selected district hospitals for the study. The pilot test outcome was satisfactory. The individual face-to-face in-depth interview guide contained questions that explored and described the challenges faced by midwives in the implementation of FBMDR in selected district hospitals. The questions were open-ended to allow the midwives to describe their in-depth views, opinions, experiences, and feelings on the phenomenon under study and allowed participants to explore and discuss their experiences, views, opinions, and recommendations about possible solutions and facilitators to midwives’ contribution to the implementation of maternal death reviews in district hospitals in the country during focus group discussions.

The matrons’ offices and conference rooms at the hospitals were used for face-to-face interviews and focus group discussions respectively. Disruptions were minimized by a door poster ‘Don’t disturb’. The researcher conducted both the individual face-to-face interviews each lasting not less than 60 min and focus group discussions between 60 and 90 min in English as per participants’ preference with the help of a trained research assistant who helped with the organization of participants, time keeping and also taking notes of some activities that were taking place during the interviews and discussions. Consent was granted by participants to have the interviews and FGDs audio-recorded. Consent forms were signed by those who volunteered to participate in the study after they were informed of the details of the study. Participants were also informed of their freedom to discontinue the study if they felt so at any point during the study. Comprehensive field notes were taken for non-verbal communication to supplement recorded data. Each interview and FGD were transcribed verbatim within 72 h after data collection with the view to go back to participants for clarity or for any missing information, where necessary, while the information was still fresh in their minds.

As a qualitative researcher, you are part of the research process & your prior experiences, assumptions, and beliefs will influence the research process. Reality is co-constructed between the researcher and the researched phenomenon and shaped by individual experiences. These are epistemological assumptions on how reality is known [24]. The researcher in the study used her experience as a midwife who once participated in maternal death reviews to understand her participant’s views, opinions, and experiences in their contribution to the implementation of FBMDRs and helped co-constructed realities together. However, was mindful and avoided biased approaches during data collection by maintaining a neutral stance while prompting and guiding the discussions. This is how reflexivity was maintained in the study.

Practice development principles were used as a framework to guide this stage of data collection. The decision was based on the theoretical underpinning of practice development. which assumes that social structures and collective culture shape the community and society in which we exist. Within the nursing and midwifery and healthcare system in general, there are cultures that either “facilitate” or “impede” participation from health professionals and patients to discuss and, in turn, make decisions to improve patient care [25]. McSherry and Warr [26] and Manley and McCormack [27], however, argued that practice development allows all participants to have an equal opportunity to participate in meaningful conversations about practice and environment thereby understanding, challenging and changing the culture to improve patient care. Thus, practitioners become active participants in knowledge generation and the continuous change process leading to embedded cultural change.

### Data management and analysis

All the generated data were manually analyzed using a thematic content analysis procedure. Five steps described by Creswell and Creswell [24] were used, which include: transcribing data, developing a category scheme, coding data, constructing and defining the themes, and presenting themes. Data was then coded and categories were created from coded data. Patterns were identified that were used to create subthemes. Finally, similar sub-themes were grouped keeping each with corresponding significant statements. The main themes were then constructed and defined after collapsing the subthemes. Deductive reasoning was used to search for units of information with similar content or meanings as well as for differences between the content of the main themes [28]. The themes were later scrutinised to determine if they formed a coherent pattern and were labelled based on the content in which they emerged in relation to FBMDR. Lastly, each theme was presented with a brief narrative description supported by the related sub-theme extracts from the data of which each was given a code consisting of an alphabetical letter and a numerical number for identification. The individual face-to-face interviews were coded with the letters “ID,” while focus group discussions were coded with the letters “FGD'' followed by numerical numbers. A personal laptop was used to capture the above transcripts into a Microsoft Excel spreadsheet. Trustworthiness of data was ensured by following the methodology for data collection, analysis and reporting.

### Ethical approval

Approval to conduct the study was obtained from the Health Studies Research Ethics Committee of the University of South Africa and the National Health Sciences Research Committee of Malawi. Eligible participants were assured of confidentiality, their participation was voluntary and they were free to discontinue the interview without any repercussion at any time. All eligible, willing and consenting participants signed the informed consent forms to participate in the study. Permission was also obtained from District Health Officers and Matrons from selected district hospitals for data collection.

## Results

A total of twenty-four Registered Nurse Midwives participated in the study of whom 12 were in senior managerial positions (two from each of the six selected district hospitals) and 12 registered nurses. In addition, 16 Nurse Midwife Technicians participated in five focus group discussions to discuss the challenges they face, possible solutions and facilitators to enhance their contribution to the implementation of FBMDR.

Table 1 above, highlights the demographic characteristics of study participants. The majority of the study participants were females married and with > 5yrs experience. All the participating facilities we funded by the government.

**Table 1:** Distribution of participants' characteristics

Characteristics	Group	Frequency (*N* = 40)	Percentage (%)
Gender	Male	8	20%
	Female	32	80%
Age	20–30	25	62.5%
	31- 44	12	30%
	45 +	3	7.5%
Educational Level	Bachelor’s Degree	24	60%
	Diploma in Nursing	16	40%
Marital Status	Married	36	90%
	Single	4	10%
Work Experience	≤ 5 years	12	30%
	5–10 years	18	45%
	11 + years	10	25%
Participants Location	Northern	12	30%
	Central	15	37.5%
	Southern	13	32.5
Sites	Urban	20	50%
	Rural	20	50%
Number of participants	Interview	12	30%
	FGD	28	70%

### Study themes

The four major challenges which emerged from the study were: i) Knowledge and skill gaps, ii) Leadership and accountability gap iii) Institutional political will and iv) Inconsistencies in the conduct of FBMDR and the possible solutions which include knowledge and skill updates, supportive supervision, and effective leadership, effective and efficient interdisciplinary working spirit, availability of material and human resources will be discussed in the section. The section below will describe the four major challenges faced by midwives in the implementation of FBMDRs:

### Knowledge and skills gaps

Inadequate knowledge and skills among the midwives were captured in this study. A majority of Nurse Midwife Technicians raised concerns about how knowledge and skills gaps limited them in performing to their expected capacity to effectively contribute to the implementation of FBMDRs. They expressed that they were not competent in data collection, data analysis, formulating diagnosis and action plans as well as the implementation of recommendations, monitoring and evaluating outcomes. Senior Registered Nurse Midwives, who participated in face-to-face interviews agreed that these knowledge and skill gaps for Nurse Midwife Technicians were hindering their effectiveness at all stages of the FBMDR process and needed urgent redress to improve their practice towards the delivery of quality maternal healthcare and contribute to the reduction in maternal death.

Factors identified as contributing to the knowledge and skills gap were identified: Lack of adequate and in-depth orientation and training on how to conduct FBMDR to midwives before they could be allocated to maternal health care units where they are expected to participate effectively in the review process and make a difference in maternal outcomes. This also deprived them of their opportunity to be updated on current best practices to continuously develop their profession. It was also revealed that the lack of mentors and supervision by senior staff and those with adequate experience affected their ability to master the necessary skills required for the reviews. Lack of access to current evidence-based information was also reported by participants as a barrier to the implementation of recommendations which led them to continue with old practices with outdated scientific bases. Both Registered Nurse Midwives and Nurse Midwife Technicians expressed that FBMDR was an on-the-job learning initiative beyond their academic preparation and scope of practice, as such they needed adequate preparation. These challenges undermined the midwives’ competencies and confidence in contributing to the implementation of FBMDR at each stage of the review process. They expressed that their contribution to FBMDRs was negligible to making a positive impact on contributing to improving the quality of maternal health care services and reduction in maternal death.

“You know (pose), the lack of knowledge among midwives regarding maternal death is a serious problem affecting our contributions to FBMDR. I have noticed that some midwives serving on the committee, mostly the low cadres, don't even know when a death of a woman can be classified as maternal death or not. If you don’t know what constitutes a maternal death, you will not even know what type of information you need to collect to confirm maternal death?” (ID 2).

In addition, lack of knowledge was evidenced by substandard information obtained during data collection which revealed: missing important information, incomplete, inaccurate, illegible, or incorrect capture of patients’ records documentation, which hindered FBMDR reviewers from fully understanding circumstances leading to a woman’s death.

“*The challenge in using the midwives’ records is mainly (due to the) poor record of data. It starts with illegible handwriting, omissions of patient personal information, information recorded without dates, time and signature; too scanty information about patients’ health passports or poorly recorded on the labour chart or no records at all. Even the entry in hospital records like registers of admissions, antenatal records, death certificates, you find that there is inadequate information or nothing documented.” *(ID 3).

Furthermore, both Registered Nurse Midwives and Nurse Midwife Technicians pointed to the need for bridging their knowledge and skills gaps. The Registered Nurse Midwives and Nurse Midwife Technicians viewed adequate orientation and training in FBMDR as an enhancer for their effective participation and contribution to the success of the reviews. The Registered Nurse Midwives explained that due to the ever-changing needs of clients, evolving and dynamic midwifery practice, new technologies, advances in research activities and demand for professional growth, they require sustainable ongoing needs. Evidence-based in-service training, workshops, Continuous Professional Development (CPD), refresher courses, upgrading qualifications and exposure to current and best practice are critical for midwives to remain relevant and responsive to the current needs in the healthcare system.

“With the dynamic nature of our profession and the ever-changing health problems experienced by women, it is impossible for us (referring to midwives) to provide quality midwifery care that responds to those needs without continuously updating our knowledge. Oh yes, we need to keep abreast of new trends in midwifery as skilled birth attendants, we are expected to have adequate knowledge and skills and competencies to provide midwifery care to both low and high-risk conditions’ (FGD2).

The need to continuously update knowledge to remain relevant and able to respond effectively to complex maternal health problems was best illustrated with the extract below.

*“As skilled birth attendants, we are expected to have adequate knowledge and skills and competencies to provide midwifery care to both low and high-risk conditions. We cannot rely only on the knowledge acquired during our formal training because nursing is always changing. We need to continuously update our knowledge to remain relevant” *(FGD6).

The midwives also narrated that sustained knowledge updates have great potential to effectively improve their confidence, critical thinking abilities, and analytical and problem-solving skills necessary for making sound, autonomous, ethical, professional and clinical decisions to advance their contribution to the reviews and improve the quality of midwifery practices.

### Leadership gap and lack of accountability

Midwives viewed the FBMDR process as a great learning opportunity for them; however, they expressed their demoralization and demotivation to participate in the reviews because of the culture of blame from senior midwifery staff and other members of the multidisciplinary team, including the District Nursing Officers, District Medical Officers and administrators, who belong to the District Health Management Team.

*“I was excited to join the review committee because I personally viewed it as a good learning opportunity. But (pose), it is frustrating and demotivating to observe that these meetings have become opportunities for certain members of the health team to accuse individuals of being responsible for the death of the woman.” *(ID 7).

Furthermore, blame-shifting was rampant, for example, clinicians and managers would blame midwives for maternal death. On the other hand, the midwives would blame their managers, clinicians and members from other departments at the facility that plays a role in the management of the women under their care. The culture of accusing team members created an unhealthy and unconducive environment to deliver collaborative and quality healthcare as a team. This led to feelings of fear of punishment, unsafe and legal implications amongst Registered Nurse Midwives and Nurse Midwife Technicians. The midwives viewed that those in leadership lacked team building and collaborative skills, did not lead by example and lacked accountability:

“Clinicians most of the time blame midwives for being responsible for maternal complications and deaths. While the midwives place their blame on clinicians and other departments like laboratory, pharmacy, transport and switch board for not acting quickly, I think these differences can amicably be attended to through the unifying responses from the managers at the facility which is generally not forthcoming hence leading to divisions and lack of professional collaboration…” (ID 10).

To address this challenge, midwives proposed improvement in communication skills and effective interpersonal relationships between members of the multidisciplinary team, effective and efficient leadership skills to create a conducive atmosphere for members to collaborate and work better as a team and towards one goal of providing quality FBMDR review process towards reducing maternal deaths and improving maternal health outcomes.

*“Interpersonal skills are important for us in the implementation of maternal death review. It involves working in a multidisciplinary team. So, we need the ability to work within the team approach and good communication skills to mutually benefit from the inputs of the other members of the multidisciplinary team without undermining the efforts of the other as this increases the likelihood of the successful implementation of our interventions” *(FGD 1).

Effective interpersonal skills as determinants of quality midwifery care were reflected in the extract below:

*“Working within a multidisciplinary team requires respect for each other, good communication and interaction with other members. Those skills will allow us to get the collaboration and support of other members in the implementation of the recommendations related to midwifery practice” *(FGD3).

Lack of supportive supervision from members of the District Health Management team was identified as one of the obstacles to midwives’ effective contribution to the implementation of FBMDR in this study. Regular supportive supervision by senior and more qualified professionals was associated with the professional development of junior staff, motivation and quality output. It was also revealed that regular supportive supervision was associated with compliance with the prescribed time and conduct of the reviews, including the execution of the recommended actions because the leaders become part of the process.

“For us to work effectively and efficiently we require regular and timely supportive supervision from our line managers” (FGD4).

### Lack of institutional political will

The inability to implement institutional policies to improve the quality of maternal healthcare due to a weak system and negative attitude was identified as a hindrance to the midwives’ contribution to the implementation of FBMDR. They pointed out that the success and failure of the initiative are determined by the institutional political will, governance and skillful leadership to timeously mobilise needed financial, human and material resources. Midwives in the study identified a lack of prioritization in addressing identified gaps from the reviews and honouring recommendations from senior staff management positions and the facility as impeding midwives’ successful contribution to the implementation of FBMDR. Leaders, including the District Nursing Officer, Medical Doctors and administrators were described as very slow to take action to address the challenges of inadequate human and material resources for effective, efficient and timely implementation of FBMDR recommended actions. The excuses given by these leaders included: a lack of sufficient budgetary allocation and a lack of prioritising midwives’ needs. Midwives explained that maternal health issues should receive priority attention to reduce preventable or avoidable deaths with cost-effective measures which are constantly overlooked. They suggested the need for those in leadership positions to be proactive in supporting midwives in their contributions to the implementation of maternal death reviews.

*We cannot say more, we need a midwifery leadership that is able to timeously facilitate the mobilisation of resources to improve our contributions to the implementation of maternal death review. It is impossible for us to be effective and efficient if we don’t have adequate material and human resources *(ID2)”

*“Because of staff shortages, most of the time midwives, mostly those working at health centres, do not attend the review meetings. You know, most of our maternal death cases occur at the health centres, which in most cases function with only one staff member who cannot leave the centre to attend the review meetings. It makes it difficult for the committee to get comprehensive information about circumstances surrounding the MD” *(ID4).

*“Most problems that we identified as avoidable causes of maternal deaths, are related to lack of material resources. For example, lack of drugs in the pharmacy, a lack of blood for transfusion, lack of equipment like blood pressures machines, thermometers which are cheap and can save the lives of women but when we ask the administration to procure them for us, they tell us they do not have adequate finances” *(ID 9).

“*We cannot say more, we need a midwifery leadership that is able to mobilise resources to improve our contributions to the implementation of maternal death review. It is impossible for us to be effective if we don’t have adequate material and human resources” (FGD 5).*

*“Management team must set their budget priorities right so that critical supplies and services for saving women's lives and improving the quality of midwifery practice are at the heart of our management team members *(FGD 2)”.

### Lack of consistency in conducting FBMDR

The study unveiled practices in the conduct of FBMDR contrary to the stipulated guidelines and procedures. Maternal deaths in many facilities where the study took place are not consistently reviewed within 72 h as per the prescribed time frame. Poor attendance and frequent cancellation of the review meetings leading to poor quality FBMDR outcomes. It was also revealed that in many cases midwives from health centres do not turn up during the reviews to give their side of the story on how a maternal death occurred. This impacts the authenticity of the collected data, hence incomplete perspectives on circumstances leading to women’s deaths. The midwives viewed these inconsistencies as contributing negatively to the quality and outcome of the reviews, hence defeating the purpose of conducting reviews to learn from the previous avoidable circumstances that led to the women’s deaths. These inconsistencies attributable to chronic staff shortages, lack of supportive supervision from senior staff members to those under their jurisdiction, increased workloads and lack of interest to attend the reviews, were highlighted during the study:

*“We expected maternal death review to follow a systematic and rigorous process to generate the best evidence to inform the diagnosis and recommendations. I am afraid to say that this is not the case in our facility. It does not motivate one to consider their recommendations… you know, the national reproductive health policy recommends that the FBMDR should be conducted within 72 hours after a maternal death has occurred. But the reality on the ground is that sometimes it takes weeks, months and in the worst cases up to a year before a maternal death case is reviewed. It is the same trend with our scheduled meetings, which are often postponed or cancelled on short notice and sometimes without notice” *(ID 4).

*“At the same time, we eventually have a backlog of documents to be reviewed, which unnecessarily increases our workload during the FBMDR. The backlog pushes the committee to rush the reviews resulting in a shady job, missing out on many important issues that could be useful in addressing the problems at hand” *(ID 11).

Regular supportive supervision from senior members of the management team was associated with the improvement in the effectiveness, efficiency and consistency in the performance of the activities related to the implementation of maternal death review at the facility. The midwives expected their seniors to offer the needed support and act as role models in the performance of activities related to FBMDR. They viewed supportive leadership as being proactive in identifying both human and material resources required for midwives to successfully contribute to the implementation of the FBMDR. Midwives also expected their leaders to offer valuable support by regularly participating in the reviews. They look up to the senior staff as role models, to coach and mentor them. The participants in the rural facilities expressed that they were least supported than their counterparts in the urban facilities due to gross staff shortages of midwives in senior positions.

*“Regular supportive supervision with senior personnel and the district health management team will provide us with the much-needed guidance on some issues that require their input. It will also motivate the committee to comply with the prescribed time frame for the reviews and facilitate the implementation of the plan of action as problems will be identified quickly and the required resources will be made available timeously. This is also more important for health centres in remote areas, where you often have one nurse-midwife technician providing care and has no senior staff to supervise him/her/” *(FGD 2).

*“It is important that senior staff like the doctor and matron with experience in FBMDR support us throughout the process. They will build capacity and help us with analytical skills on how to conduct the review and how to generate solutions. When senior staff attend the reviews, we see that the reviews are taken seriously and good ideas come out of it” *(FGD 7).

Midwives argued that regular supportive supervision is an indicator of the commitment of the leadership to the maternal death review process. This perceived commitment will be translated to the high attendance of the review meetings by members of the multidisciplinary team.

*“We have noted high attendance of review meetings every time people are informed of the attendance of district management teams and other senior members of the multidisciplinary team. It also shows their commitment to the review process and consequently high chances of getting the support required for the recommendations” *(FGD4).

Supportive supervision was reported to increase their confidence, critical thinking and problem-solving skills.

*“When you are visited regularly by senior staff, you will have the opportunity to raise some of the burning issues directly with them and get guidance on the spot. It makes a big difference for staff motivation” *(FGD2).

## Discussion

This study aimed at establishing the challenges faced by district-based midwives in the implementation of FBMDR in Malawi. Four major findings emerged: i) inadequate knowledge and skills gap, ii) leadership gap and lack of accountability, iii) lack of institutional political will and iv) lack of consistency in conducting FBMDR. This study has provided insight on challenges faced by midwives that impede them from effectively contributing to the implementation of maternal death review at district hospitals, which is an initiative aimed to improve the quality of maternal healthcare and reduce the currently high maternal mortality rates by qualitatively learning from previous experiences of maternal deaths, understanding causes and circumstances leading to mothers dying during pregnancy and possible solutions to prevent or avoid its occurrence. The section below provides a discussion of these challenges and possible solutions:

### Knowledge and skills gap

In this study, inadequate knowledge and skills gaps were found to have impeded the midwives’ contributions to the implementation of FBMDR. Midwives reported that they were limited in effectively administering their duties of FBMDR at each of the five stages of the process that include: case identification, and notification, data collection, analysis of findings, implementation of recommended actions, follow up and evaluation [11]. Worrisomely, midwives who are frontline maternal healthcare providers do not have the necessary competencies to participate in the facility’s maternal death reviews comprehensively and effectively. Skilled attendants are key in the fight against all preventable and avoidable maternal morbidity and mortalities. The presence of skilled professionals (doctor, midwife or nurse) during delivery is crucial in reducing maternal and neonatal deaths. In 2010 approximately 287,000 women died while pregnant or giving birth and 3.1million newborns died in the neonatal period [1]. Skilled care at every birth can serve the lives of women and newborns [1]. Midwives participate in safe motherhood initiatives through the provision of basic and comprehensive emergency obstetric and neonatal care, and maternal neonatal and reproductive health services [29, 30].

A study in Kenya, Nigeria and India, however, found the following challenges in the implementation of FBMDR: lack of knowledge amongst facility staff involved in FBMDR reporting processes leading to poor compliance, incomplete and inaccurate reporting and failure to dispatch case records to central committee leading to substandard review outcomes, lack of knowledge and skills were a major hindrance for midwives to successfully contribute to improving the quality of maternal health care at district hospitals. Lack of adequate pre-service training and on-the-job continuous professional development were blamed for these challenges [20]. Also, a lack of orientation on FBMDR, inadequate access to personal professional development, inability to use evidence-based knowledge to inform practice and inability to facilitate clinical learning were found to impede FBMDR activities. Skill-based training was found to be important for healthcare providers, increased competency in skilled birth attendance, and emergency obstetric and neonatal care. Improved knowledge and skills of healthcare providers through education, coaching, mentorship and other professional development activities were consistently identified as an important facilitator of the implementation of guidelines by the professionals while lack of capacity building was identified as a major factor contributing to the lack of knowledge and skills among midwives [31]. A study in the Ashanti region in Ghana highlighted the need to continuously support the on-the-job training capability building of midwives to enhance their contributions to the implementation of facility-based maternal death review, [32]. De Brouwere [33], found that regular evaluation of staff competencies (knowledge, attitude and skills) was associated with the smooth implementation of different activities of the facility-based maternal death review [34], on the other hand, noted that training on maternal death reviews in sub-Saharan African countries, including Malawi, was facing sustainability challenges after donor funded FBMDR training pulled out and governments could not sustain funding to scale up the training.

### Leadership gap and lack of accountability

The importance of leadership cannot be overemphasized in the healthcare delivery system. Leaders and managers need to lead with honesty and integrity. Creating a culture of accountability in healthcare is key and must start at a personal level. Leadership can positively or negatively affect the quality-of-service delivery at an institution, for example, the effects it might have on the stress or wellbeing of the staff which in turn is related to the poor quality of care produced [35]. This study identified ineffective leadership as one of the challenges hindering midwives’ successful contribution to the implementation of FBMDR. The culture of blaming midwives’ staff by those in leadership and administration at the facilities increased their stress levels and robbed them of their confidence and freedom to participate and contribute during review meetings. Insufficient competencies in the review process were cited as the main reason for this blame by their seniors [35]. The study further found that apportioning of blame led to frustration, demotivation, sense of victimisation, stress and fear of legal repercussions, which consequently led to junior midwives resenting the reviews as a result [35]. The study also noted that those in leadership did not take the necessary steps to address the problems and projected the blame on their juniors which showed a lack of accountability for results for their roles hence affecting the quality of the FBMDR process aimed at improving maternal healthcare outcomes.

 Ravichandran and Ravindran [36] highlights the importance of the ability of review team members to share diverse information, disclose errors and seek help and feedback from other teams and administration without fear of punitive measures by senior staff and leaders, given the abundance of evidence that medical errors are largely attributed to systems rather than individuals. A study in Kenya reports that without an adequate legal framework and sensitization of health workers to the “blame-free” principle, the government plans to progress the MDR system may stall. Because of this, a legal framework is being enforced to ensure that information obtained and retrieved as part of the Maternal Death Surveillance and Response (MDSR) process is not used for litigation and to provide reassurance to health workers of its blame-free principle of MDSR. According to McAuliffe et al. [37], Malaysia is one of the countries that conduct FBMDR as a “no shame, no blame process, with the focus on learning from the maternal deaths to improve health systems and practices. In addition, in Malaysia, it was found that the term *“substandard care”* originally used in Malaysian maternal death review system, to categorise inappropriate or deficient care, was changed to “*remediable factors*'' thereby projecting a positive image of caregivers and the care they provide. McAuliffe et al. [37] in their studies emphasised that many contributing factors to maternal death are beyond the control of an individual. A study in Tanzania, Malawi and Mozambique found that ongoing support is needed for health workers in the frontline services delivery to perform to their full potential and deliver quality care [38]. Adding that, when healthcare workers receive formal supervision, their participation increase in a more open and inclusive environment which provides space for them to express ideas and be heard. The study further supports the need to strengthen leadership in the implementation of the framework.

### Lack of consistency in conducting FBMDR

Nonadherence and compliance with prescribed review procedures and standards were identified as some of the barriers faced by midwives in implementing FBMD. Lack of consistency in the timing and conduct of the reviews and low attendance beyond the minimum expected quorum for review meetings were due to staff shortages, lack of periodization of the FBMDR activities, and lack of commitment by leaders and staff since there were no incentives. Midwives reported demotivation including lost opportunities to learn from causes and circumstances surrounding maternal deaths. Postponement of the reviews increased the workload due to the backlog of unreviewed cases of maternal deaths and generally poor quality of recommendations generated from rushed reviews leading to repeating the same fetal mistakes that claim the lives of mothers. Timeous reviews are crucial in improving maternal health care and reducing maternal deaths. Unless there is improvement in individual capabilities, leadership, support structures and resources, midwives will continue to be ineffective and inefficient in their timely contribution to the implementation of the reviews. Ministry of Health Malawi [13], de Brouwere et al. [33] and  Combs Thorsen et al. [38] proposed that activities of FBMDR should be guided by protocols set by the Ministry of Health based on the World Health Organisation guidelines.

The multidisciplinary FBMDR committees are expected to function according to the set standards, protocol and sense of responsibility of its members [19, 39]. The multidisciplinary FBMDR committees assess every case of maternal death that occurred in the facility with the view of identifying the probable cause of death, the preventable conditions and contributing factors [19, 39]. Members of these multidiscipline committees include the District Health Management Team, obstetricians, medical officers, anaesthetists, midwives and other senior personnel. They conduct the reviews as part of their professional development with no expectation of extra pay [19].  However, in most countries where the FMDR are being implemented, the absence of the members from the senior clinical staff and the District Health Management Team members in the review meetings, the lack of support from managers and insufficient resources emerged as major obstacles to the performance of the FBMDR committees [19, 39]. On the other hand, the establishment of a central national administrative support system, the availability of support structures and policies, training  and resources (financial, human and materials) ensured smooth performance of the FMDR committees in countries such as Malaysia, the Republic of South Africa and the United Kingdom [40].

Good leadership, coordination of training of all key staff, staff members’ positive attitude towards the implementation of the review, and the availability of external support are viewed as major facilitators of the implementation of the reviews [41]. Administrative support, positive attitudes, and active nurse and physician champions were also found to be facilitators of the successful implementation of all the activities of the project, while negative attitudes, lack of support from managers, and lack of champions were identified as barriers to the implementation of the activities of the project, leading to demotivation of staff members, [41]. For the FBMDR cycle to work efficiently as a continuous quality improvement process to prevent future maternal deaths, certain inputs and processes need to be in place at a health facility, district and national level [41]. In Malawi the FBMDR is guided by set procedures, standards and guidelines by the Ministry of Health, Sexual Reproductive Health Unit, however, committed leadership is required to ensure systems are in place for successful review outcomes [13].

## Recommendations

To effectively contribute to the implementation of FBMDRs, midwives at different positions in their profession need to be committed to playing their roles and responsibilities with diligence. Those in senior positions like nursing officers and matrons should ensure that they offer leadership, management, supportive supervision, resource mobilisation, and plan and implement capacity building for midwives as it relates to the need for the efficient conduct of reviews. They should desist from blaming junior staff for maternal deaths, instead mutually identify avoidable problems and together identify strategies that will help to improve the quality of maternal health services. The need to promote a high level of multidisciplinary team spirit and to lead by example through active participation in the review. They need to time and again advocate midwives at the facility as well as those in the health centres need for example ensure the availability of both material and human resources. Organise in-service training and refresher course. The bedside midwives should ensure that they have the necessary competencies to participate in the reviews. They need to build their capacities through continuous professional development and practice development strategies for them to effectively contribute to maternal death reviews.

These challenges and recommended solutions are a blueprint for designing strategies that are specifically tailored towards improving and developing midwives practice for them to successfully implementation of FBMDR and consequently contribute to reduction of avoidable future maternal deaths. This approach helped the researcher to develop practice development strategies for district-based midwives in the implementation of FBMDR. The researcher argued that effective support strategies should be based on midwives’ work experiences in the context of the healthcare delivery system of Malawi. Secondly, the researcher adds that midwives have different experiences which would be best depicted within the constructivist worldview.

## Conclusion

This study has illuminated the challenges faced by midwives as they participate in the implementation of the FBMDR and its related activities. The study findings included: i) inadequate knowledge and skills gap, ii) the blame game, iii) lack of prioritization in addressing gaps and honouring recommendations and iv) lack of consistency in conducting FBMDR at all levels of the healthcare system as some of the main barriers for midwives to successfully participate in the reviews. The study has also highlighted solutions to address the identified challenges. These findings provided a contextual basis to inform the design of support strategies for the practice development of district-based midwives in the context of the healthcare system in Malawi. This framework will emphasise the strengthening of the healthcare system with a specific focus on the government's responsibility to address the challenges related to service delivery, health workforce, health information systems, access to essential health medicines financing, leadership and governance at all levels of the healthcare delivery system (policy, program planning and provider level) concerning WHO health system building blocks. Addressing the challenges faced by midwives calls for consented efforts and commitment from all stakeholders including government, Public and Private Partnerships, civil society, Non-Government Organisations, local midwifery associations and regulatory bodies, bilateral organisations and the community. The implementation process could be undertaken through scaling up sound training and policies that would redress accountability and leadership issues and strengthen ongoing monitoring and evaluation of FBMDR at each stage of the review process.

## Limitations

Due to the shortage of midwives in the country, it was difficult to get enough participants. Some district hospitals could not be accessible due to geographical position. All these limitations made it difficult to achieve the sample size of participants where data could be collected to generalise the findings across the country. To overcome these limitations, the two categories of midwives were included in the study to maximise data and get diverse views. Triangulation of data collecting methods was also regarded as a measure to litigate those limitations.

## Data Availability

All the data sets used during the study are available in the manuscript.

## Abbreviations

CPD: Continuous Professional Development
FBMDR: Facility Based Maternal Death Review
LMIC: Low Middle-Income Countries
MDSR: Maternal death Surveillance and Response
MDG: Millennium Development Goals
SDG: Sustainable Development Goals
FGD: Focus Group Discussion
WHO: World Health Organisation
